# Different neural codes result in bidirectional connectivity formed by the same model of spike-timing-dependent plasticity

**DOI:** 10.1186/1471-2202-12-S1-P247

**Published:** 2011-07-18

**Authors:** Florian Hauser, Andreas Knoblauch, Günther Palm

**Affiliations:** 1 Institute of Neural Information Processing, Ulm University, 89069 Ulm, Germany; 2 Honda Research Institute EU, 63073 Offenbach/Main, Germany

## 

Recent studies on phenomenological models of spike-timing-dependent plasticity (STDP) extend the idea of a pure spike-based paradigm to the level of dendritic voltage [1]. In such models, the decision to induce early long-term potentiation (eLTP) or long-term depression (eLTD) not solely depends on the time difference between pre- and post-synaptic spikes but also on the precise dynamics of the post-synaptic potential. From simulations of small neural networks and different stimulation schemes corresponding to different neural codes, these studies have concluded that the pattern of synaptic connectivity would directly follow from the neural code and vice versa. For example, they have suggested that strong uni-directional connectivity would emerge through temporal coding, whereas bidirectional connectivity would be indicative for a rate code. Relying on results from earlier studies on first generation STDP models [2-4], synchronous temporal codes forming bidirectional connectivity patterns were considered unlikely in [1]. However, recent findings revise a general exclusion of this relationship [5] even for first generation STDP models. In particular, our work shows that realistic STDP models as proposed in [1] can actually not predict the neural code (temporal vs. rate coding) by looking at the pattern of synaptic connectivity within a specific brain area. With the same kind of simulations as in [1], but additionally considering stimulation conditions for synchronous temporal coding, we show that bidirectional connections are still possible. Furthermore we discuss the influence of synchronization precision and oscillatory activity.
